# Using NU-KNIT® for hemostasis around recurrent laryngeal nerve during transthoracic esophagectomy with lymphadenectomy for esophageal cancer

**DOI:** 10.1186/1756-0500-7-127

**Published:** 2014-03-06

**Authors:** Yasushi Rino, Norio Yukawa, Tsutomu Sato, Naoto Yamamoto, Hiroshi Tamagawa, Shinichi Hasegawa, Takashi Oshima, Takaki Yoshikawa, Munetaka Masuda, Toshio Imada

**Affiliations:** 1 Department of Surgery, Yokohama City University, Yokohama, Japan; 2 Gastroenterological Center, Medical Center, Yokohama City University, Yokohama, Japan; 3 Department of Gastrointestinal Surgery, Kanagawa Cancer Center, Yokohama, Japan; 4 Saiseikai Yokohama Nanbu Hospital, Yokohama, Japan

## Abstract

**Background:**

We thought that using electrocautery for hemostasis caused recurrent laryngeal nerve palsy. We reflected the prolonged use of electrocautery and employed NU-KNIT® to achieve hemostasis nearby the recurrent laryngeal nerve. We assessed that using NU-KNIT® hemostasis prevented or not postoperative recurrent laryngeal nerve palsy, retrospectively. The present study was evaluated to compare using electrocautery hemostasis with using NU-KNIT® hemostasis during lymphadenectomy along recurrent laryngeal nerve. The variables compared were morbidity rate of recurrent laryngeal nerve palsy, operation time, and blood loss.

**Results:**

We use NU-KNIT® to achieve hemostasis without strong compression. This group is named group N. On the other hand, we use electrocautery to achieve hemostasis. This group is named group E. Complication rate of recurrent laryngeal nerve palsy was higher in group E (55.6%) than group N (5.3%) (p = 0.007).

**Conclusions:**

Even hemostasis using NU-KNIT® was slightly more time-consuming than using electrocautery, we concluded that it would be useful to prevent recurrent laryngeal nerve palsy.

## Background

Surgery for transthoracic esophagectomy with lymphadenectomy is still associated with high complication rates of recurrent laryngeal nerve palsy [1,2] and a long-standing negative impact on the patient’s quality of life (QOL). In our institute, the rate of recurrent laryngeal nerve palsy was 13.3% after esophagectomy with lymphadenectomy for thoracic esophageal cancer until June 2008. Because of the high rate of #106 rec R and L lymph nodes with cancer involvement, complete lymphadenectomy along bilateral recurrent laryngeal nerve is desirable, but it often cause recurrent laryngeal nerve palsy leading dysphagia or aspiration pneumonia. Thus, reducing the risk of such complications could reduce the negative impact on QOL after esophageal cancer surgery. Electrocautery is a safe and effective method of hemostasis during esophageal cancer surgery. There are no absolute contraindications to electrosurgery. Each electrocautery device can deliver heat at a single temperature or range of temperatures, between 100°C and 1200°C. We assumed that the temperature using electrocautery for hemostasis caused recurrent laryngeal nerve palsy and reflected the prolonged use of electrocautery and employed NU-KNIT® [3,4], in January 2010, to get hemostasis nearby the recurrent laryngeal nerve. NU-KNIT® has 3 times more dense than SURGICEL® Original Absorbable Hemostat >36% faster time to hemostasis. Dense knit material provides strength in the presence of heavy bleeding. NU-KNIT® is soft, pliable weave designed to hold a suture and for placement on delicate tissue [3,4]. Indications of SURGICEL is that haemostat is used adjunctively in surgical procedures to assist in the control of capillary, venous, and small arterial haemorrhage when ligation or other conventional methods of control are impractical or ineffective [4]. We assessed that using NU-KNIT® hemostasis prevented or not postoperative recurrent laryngeal nerve palsy, retrospectively. The present study was evaluated to compare using electrocautery hemostasis with using NU-KNIT® hemostasis during lymphadenectomy along recurrent laryngeal nerve. The variables compared were morbidity rate of recurrent laryngeal nerve palsy, operation time, and blood loss.

## Methods

The patients were candidates for an esophagectomy at our institute from July 2008 to August 2011. The patients are consecutive and non-selected. This study was performed with the approval of an appropriate ethics committee. Informed consent was obtained from all patients to use NU-KNIT for this study according to the institutional rules of the Yokohama City University Hospital. The study protocol conforms to the ethical guidelines of the 1975 declaration of Helsinki as reflected in a priori approval by the institution’s human research committee.

Between January 2010 and August 2011, we use NU-KNIT® to achieve hemostasis without strong compression. This group is named group N (mean age: 67.6 years; age range: 56–76 years; 18 men, 1 woman). On the other hand, except the preceding period, we use electrocautery to achieve hemostasis. This group is named group E (mean age: 65.2 years; age range: 49–75 years; 8 men, 1 woman) (Table 1).

**Table 1:** Patients characters and operation datas

**Hemostasis**	**Sex**	**Age**	**pTNM stage**	**Blood**	**Operation time/**	**Recurrent nerve**
**methods**	**(M/F)**	**(y/o)**		**loss (ml)**	**thoracotomy time (min)**	**palsy (y/n)**
Group E	M	75	pT1bN0M0 I	1249	703/286	y (bilateral)
	M	67	pT3N1M0 III	1343	500/186	n
	M	64	pT3N0M0 II	669	503/198	y
	M	57	pT1bN0M0 III	389	518/212	y (bilateral)
	M	49	pT4N0M1 IVb	200	379/153	n
	M	51	pT3N1M0 III	100	395/210	n
	M	60	pT1bN1M0 II	500	476/160	y
	M	61	pT3N2M0 III	600	502/235	y
	F	57	pT3N0M0 II	208	640/160	n
Average ± S.D.		60.1 ± 8.0		584.2 ± 446.4	512.9 ± 103.6/200.0 ± 42.5	Complication rate; 55.6%*
Group N	M	63	pT2N1M0 II	795	594/220	n
	M	61	pT2N0M0 II	3000	660/240	y
	M	67	pT3N0M1 IVb	555	429/190	n
	M	66	pT1aN0M0 0	500	499/170	n
	M	68	pT3N1M0 III	691	590/235	n
	M	66	pT2N2M0 III	2140	513/199	n
	M	64	pT3N0M0 II	250	545/264	n
	M	70	pT1aN0M0 0	765	558/281	n
	M	76	pT2N0M0 II	2992	472/239	n
	M	63	pT1bN0M0 I	788	623/353	n
	M	72	pT3N1M0 III	278	466/202	n
	M	74	pT1bN0M0 I	300	561/324	n
	F	74	pT2N0M0 II	675	355/236	n
	M	56	pT3N2M0 III	100	435/195	n
	M	74	pT1aN0M0 0	319	541/284	n
	M	63	pT1bN0M0 I	400	508/272	n
	M	69	pT3N1M0 III	757	621/273	n
	M	75	pT1bN0M0 I	341	708/374	n
	M	63	pT1bN0M0 I	203	618/351	n
Average ± S.D		67.6 ± 5.6		834.2 ± 877.9	541.9 ± 88.0/258.0 ± 59.3	Complication rate; 5.3%

Group E; using electrocautery to achieve hemostasis, Group N; using NU-KNIT® to achieve hemostasis.

M; male, F; female, S.D.; standard deviation, *; p = 0.007.

All esophagectomy with lymphadenectomy were performed by same surgeon.

We investigated the rate of recurrent laryngeal nerve palsy, operation time, and blood loss in patients who underwent esophagectomy with lymphadenectomy, retrospectively. Recurrent laryngeal nerve palsy was diagnosed by otolaryngologists.

Statistical analysis was conducted using Student’s *t*-test and *χ*^2^ test with *P* < 0.05 taken as significant.

## Results

The results are summarized in the Table 1. In group E, five of 9 cases (55.6%) were complicated recurrent laryngeal nerve palsy. Two of 5 recurrent laryngeal nerve palsy cases were bilateral. Four of 5 recurrent laryngeal nerve palsy patient in group E were reversible. Mean blood loss was 584.2 ml. Mean operation time was 521.9 min and mean thoracotomy time was 200.0 min. In group N, one of 19 cases (5.3%) was complicated recurrent laryngeal nerve palsy. There is no bilateral recurrent laryngeal nerve palsy. Mean blood loss was 834.2 ml. Mean operation time was 541.9 min and mean thoracotomy time was 258.0 min. Three of 11 cases were lost blood more than 1,000 ml with injured spleen. Mean blood loss was 482.3 ml except for the three cases. There were no significant difference between two groups in the incidence of complications; blood loss, and operation time. But the incidence of complication of recurrent laryngeal nerve palsy was significantly higher in group E (55.6%) than group N (5.3%) (p = 0.007).

## Discussion

In our institute, the rate of recurrent laryngeal nerve palsy was 13.3% after esophagectomy with lymphadenectomy for thoracic esophageal cancer until June 2008. Okuyama et al. reported that they compared the outcomes of cervical hand-sewn anastomosis and intrathoracic stapled anastomosis performed after esophagectomy and gastric reconstruction. The respective rates of recurrent laryngeal nerve palsy were 38.8% versus 7.1% (P < 0.05) [1]. Fang et al. reported that patients in the three-field lymphadenectomy experienced significantly more recurrent laryngeal nerve palsy than the two-field lymphadenectomy (22.9% *vs*. 9.6%, *P* = 0.089) [2]. The rates of recurrent laryngeal nerve palsy after esophagectomy are still high. Sometimes the time to achievement of adequate hemostasis using electrocautery was prolonged. Cauterization using electrocautery is superior to suture as a method of achieving hemostasis, with significantly less blood loss and shorter operative time [5]. Parenchymatous hemostasis using electrocautery greased with lidocaine gel was easy to perform and achieved complete hemostasis of the minor blood vessels in all patients. No postoperative bleeding occurred and the follow-up course was satisfactory. Electrocautery greased with lidocaine gel is an inexpensive, readily available, and efficient method to achieve hemostasis of minor vessels in hepatic, splenic, and bone operations [6]. However, electrocautery can deliver heat at a single temperature or range of temperatures, between 100°C and 1200°C. Physicians must consider the histologic properties of the tissue to be treated, the area and depth of destruction desired, possible recurrent laryngeal nerve palsy after esophagectomy. We assumed that the temperature using electrocautery for hemostasis caused recurrent laryngeal nerve palsy and employed NU-KNIT® [3,4]. The use of NU-KNIT® as buttress material may be effective in reducing acute postoperative bleeding in laparoscopic Roux-en-Y gastric bypass at a significantly lower cost [7]. Surgicel NU-KNIT® for the sternum bleeding permitted a good hemostatic effect without wax use [3,8]. Despite the probe of electrocautery is not used to achieve hemostasis around recurrent laryngeal or vagus nerve, complication rate of recurrent laryngeal nerve palsy was so high. We guessed that prolonged electrosurgical hemostasis time affected the nerve. We quit using electrocautery near the nerve to prevent recurrent laryngeal nerve palsy. Since January 2010, we have employed NU-KNIT® to achieve hemostasis without press the nerve. We successfully performed an esophagectomy with lymphadenectomy in 11 esophageal cancer patients using NU-KNIT® from January 2010 to December 2010. One of 11 cases complicated recurrent laryngeal nerve palsy. This case has lymph node adhesion to left recurrent laryngeal nerve. Even the left recurrent laryngeal nerve was preserved using scissors, the palsy remained.

NU-KNIT® has 3 times more dense than SURGICEL® Original Absorbable Hemostat >36% faster time to hemostasis. Dense knit material provides strength in the presence of heavy bleeding. NU-KNIT® is soft, pliable weave designed to hold a suture and for placement on delicate tissue [3,4]. Because of these characters of NU-KNIT®, we employed it to hemostasis near the nerve. Blood loss and operation time were increased than using electrocautery to hemostasis, but there were no significant difference. Thoracotomy time was also prolonged, but there were no significant difference, as well.

## Conclusions

Hemostasis using NU-KNIT® can be good strategy to prevent recurrent laryngeal nerve palsy in transthoracic esophagectomy. But more cases are needed to evaluate a confident prevention of recurrent laryngeal nerve palsy.

## Abbreviations

QOL: Quality of life; NAC: Neoadjuvant chemotherapy.

## Competing interests

The authors declare that they have no competing interests.

## Authors’ contributions

All authors carried out these procedures. YR wrote the manuscript. OT participated in paper revise. All authors read and approved the final manuscript.

## References

[B1] Okuyama M Motoyama S Suzuki H Saito R Maruyama K Ogawa J Hand-sewn cervical anastomosis versus stapled intrathoracic anastomosis after esophagectomy for middle or lower thoracic esophageal cancer: a prospective randomized controlled study Surg Today 2007 37 11 947 952 10.1007/s00595-007-3541-5 17952523

[B2] Fang WT Chen WH Chen Y Jiang Y Selective three-field lymphadenectomy for thoracic esophageal squamous carcinoma Dis Esophagus 2007 20 3 206 211 10.1111/j.1442-2050.2007.00671.x 17509116

[B3] Mair H Kaczemared I Oberhoffer M Groetzner J Daebritz S Reichart B Surgicel Nu-knit hemostat for bleeding control of fragile sternum J Thorac Cardiovasc Surg 2005 130 2 605 606 10.1016/j.jtcvs.2005.04.013 16077453

[B4] SURGICEL® Absorbable Hemostat Full Prescribing Information [http://www.ethicon360.com/products/surgicel-nu-knit-absorbable-hemostat, http://www.ethicon360emea.com/sites/default/files/products/SURGICEL_NU_KNIT_Product_Labeling_0.pdf]

[B5] Kamat AA Kramer P Soisson AP Superiority of electrocautery over the suture method for achieving cervical cone bed hemostasis Obstet Gynecol 2003 102 4 726 730 10.1016/S0029-7844(03)00622-7 14551002

[B6] Petroianu A Hemostasis of the liver, spleen, and bone achieved by electrocautery greased with lidocaine gel Surg Today 2011 41 2 300 302 10.1007/s00595-009-4211-6 21264774

[B7] Moon R Teixeira A Varnadore S Potenza K Jawad MA Reinforcing the staple line with Surgicel® Nu-knit® in Roux-en-Y gastric bypass: comparison with bovine pericardial strips Obes Surg 2013 23 788 793 10.1007/s11695-013-0898-y 23471675

[B8] Belov IV Bazylev VV Alekseev IA The use of oxygenated regenerated cellulose for hemostasis in cardiac surgery Khirurgiia (Mosk) 2009 5 10 14 19491761

